# Effect of Liraglutide on Serum TSH Levels in Patients with NAFLD and its Underlying Mechanisms

**DOI:** 10.1155/2022/1786559

**Published:** 2022-10-13

**Authors:** JiaoJiao Ye, Jing Xu, WenJie Wen, Bin Huang

**Affiliations:** ^1^Department of Infectious Diseases, The First Affiliated Hospital of USTC, Division of Life Sciences and Medicine, University of Science and Technology of China, Hefei, Anhui 230001, China; ^2^Department of Endocrinology, The First Affiliated Hospital of USTC, Division of Life Science and Medicine, University of Science and Technology of China, Hefei, Anhui 230001, China

## Abstract

This study aimed to evaluate the effect of liraglutide on serum thyroid-stimulating hormone (TSH) levels in patients with type 2 diabetes mellitus (T2DM) and explore the underlying mechanisms via bioinformatics analysis. A total of 49 obese/overweight patients with T2DM received liraglutide during outpatient visits or hospitalization in the Department of Endocrinology. Meanwhile, the control group included 49 patients with T2DM but without nonalcoholic fatty liver disease (NAFLD) who were matched for age and sex (baseline from July 2016 to June 2021). Follow-up data on the last use of liraglutide were also retrieved. Age, sex, body mass index (BMI), and duration of diabetes were obtained from the participants' records. All patients were tested for biochemical markers hemoglobin A1c (HbA1c), alanine transaminase, aspartate transaminase, free triiodothyronine, free thyroxine (FT4), and TSH at baseline and follow-up. After adjusting for all factors with a *p*-value < 0.05, BMI, HbA1c, LDL, FT4, and TSH were identified as significant independent risk factors for NAFLD in the univariate analysis. Following liraglutide therapy (average time 16 months), these patients had significantly lower BMI, HbA1c, and TSH but higher high-density lipoprotein (HDL) levels than those in the baseline data (all *p* < 0.05), and further subgroup analysis stratified by duration of liraglutide use showed that the test for time trends had statistical differences in BMI and TSH but not in HbA1c and HDL. After the therapy, the NAFLD and NASH groups showed significantly decreased TSH levels after liraglutide therapy compared with the corresponding baseline data. Furthermore, the expression of THRB, which encodes TR*β*, was significantly decreased in the NAFLD group, which may explain the thyroid hormone resistance-like manifestation in the clinical findings. In conclusion, liraglutide improves hepatic thyroid hormone resistance in T2DM with NAFLD, and restoration of impaired TR*β* expression in NAFLD is a potential mechanism involved in the process of liraglutide therapy.

## 1. Introduction

Type 2 diabetes mellitus (T2DM) is one of the largest global epidemics and is commonly associated with multiple organ dysfunction, including liver, kidney, and cardiovascular diseases [1]. Patients with T2DM have a very high incidence of nonalcoholic fatty liver disease (NAFLD) and an increased risk of adverse clinical outcomes and death [2]. However, existing clinical practice guidelines for NAFLD management provide limited recommendations for the treatment of T2DM with NAFLD. The development of glucagon-like peptide-1 receptor (GLP-1R) agonists to treat T2DM and obesity/overweight has gained increasing attention [3]. Liraglutide, a commonly used GLP-1R agonist in clinical practice, is also being investigated to treat NAFLD, a condition that is common in those with obesity and T2DM and is increasing in incidence and for which drug development is challenging and few effective therapies are available [4, 5].

The liver and thyroid are intimately linked, with thyroid hormone (TH) playing important roles in lipogenesis, cholesterol metabolism, and beta-oxidation (fatty acid oxidation) [6, 7]. In our previous study involving 369 euthyroid T2DM individuals with normal thyroid function suspected of NAFLD, we found that their levels of free triiodothyronine (FT3) and thyroid-stimulating hormone (TSH) were higher than those in individuals without NAFLD, which confirmed that these patients had TH resistance-like manifestations [8]. From the perspective of a negative feedback mechanism, this manifestation may indicate that the liver, one of the most important organs for metabolism, damages the TH signaling pathway. Whether liraglutide, as a representative drug that can reduce body weight and improve NAFLD, will alleviate thyroid resistance in the liver has not been reported. The aim of this study was to evaluate the effect of liraglutide on serum TSH levels in T2DM patients and the underlying mechanisms using bioinformatics analysis.

## 2. Materials and Methods

### 2.1. Study Population

A total of 49 patients with T2DM and NAFLD received liraglutide-initiated treatment during outpatient visits or hospitalization in the Department of Endocrinology of the First Affiliated Hospital (Anhui Provincial Hospital) of the University of Science and Technology of China. Meanwhile, 49 patients with T2DM but without NAFLD who were matched for age and sex (baseline from July 2016 to June 2021) comprised the control group. The diagnosis of NAFLD was based on abdominal ultrasonography, and the specific method of operation was consistent with our previous study [8]. All patients had a body mass index (BMI) above 24 kg/m^2^ (considering ethnic differences, our study adopted the obesity diagnostic criteria recommended by the World Health Organization for Chinese people: BMI ≥ 24 kg/m^2^ was overweight and BMI ≥ 28 kg/m^2^ was obese) and normal thyroid function (euthyroid, defined as both free thyroxine and TSH within the reference range). Follow-up data on the last use of liraglutide were retrieved, and the duration of liraglutide use was recorded. The requirement for informed consent was waived, as this was a retrospective study of data collected from medical records of the participants. We divided the 49 patients into two groups: before (liraglutide treatment was initiated) and after (follow-up data on the last use of liraglutide). The exclusion criteria were as follows: (1) patients with acute complications of diabetes; (2) abnormal thyroid function, intake of thyroxin or other thyroid-relevant drugs, or use of liver-protecting drugs; and (3) presence of cancer, liver disease (viral, autoimmune, alcoholic hepatitis, etc.), or severe chronic kidney disease (defined as eGFR ≤6 mL/min/1.73 m^2^).

### 2.2. Clinical and Laboratory Evaluation

Age, sex, BMI, duration of diabetes, and use of antidiabetic drugs (administered continuously for at least 3 months; metformin intake defined as more than 1.5 g/day) were obtained from the records at baseline. Use of antidiabetic drugs that lasted more than half the duration of liraglutide use was included in the follow-up study. All patients were tested for biochemical markers at baseline and follow-up as follows: alanine transaminase (ALT) and aspartate transaminase (AST) for liver function; creatinine for kidney function; hemoglobin A1c (HbA1c) for glucose metabolism; low-density lipoprotein cholesterol (LDL-c) and high-density lipoprotein cholesterol (HDL-c) for lipid metabolism; and FT3 (normal range: 3.28-6.47 pmol/L), free thyroxine (FT4; normal range: 7.90-19.05 pmol/L), and TSH (normal range: 0.350-4.949 mIU/L) for thyroid function.

### 2.3. Bioinformatics Analysis

The dataset GSE48452 was downloaded from the Gene Expression Omnibus (https://www.ncbi.nlm.nih.gov/geo). The samples were divided into two groups: 24 obese patients with NAFLD and 16 with healthy livers. After consolidation and normalization of the RNA-sequencing data, 118 differentially expressed genes involved in NAFLD were screened using the “limma” package (adjusted *p* < 0.05 and |logFC| > 0.5). The pathview library (https://pathview.uncc.edu/home) was used to visualize the TH signaling pathway.

### 2.4. Statistical Analysis

IBM SPSS 22.0 was used for processing, and statistical significance was set at *p* < 0.05. Continuous measurements that were normally distributed are expressed as the mean (standard deviation), while those that were not normally distributed are expressed as the median (interquartile range). Categorical variables are expressed as the frequency and percentage (%). Independent tests, including *t*-tests, chi-square tests, and Mann–Whitney *U* test, were used to compare the two groups. Logistic stepwise regression was used to determine the independent factors. In the follow-up data, the corresponding parameters before treatment were used as covariates, and the differences before and after treatment were analyzed using covariance analysis.

## 3. Results

### 3.1. Demographic and Metabolic Characteristics of Study Subjects

The data of 98 patients with T2DM (65.3% male and 51.2% with NAFLD) were evaluated. The mean age of the study subjects was 58.10 ± 12.49 years, ranging 19-84 years. The duration of diabetes ranged from 0 to 35 years. Patients in the NAFLD group had significantly higher BMI, HbA1c, LDL-c, FT4, and TSH levels and lower duration of diabetes and HDL levels than those in the non-NAFLD group (all *p* < 0.05). No significant differences in sex, age, fasting blood glucose, ALT, AST, estimated glomerular filtration rate (eGFR), FT3, or the use of hypoglycemic drugs were observed between the two groups (all *p* > 0.05; Table 1).

### 3.2. Independent Risk Factors Associated with the Incidence of NAFLD

**Figure 1:**
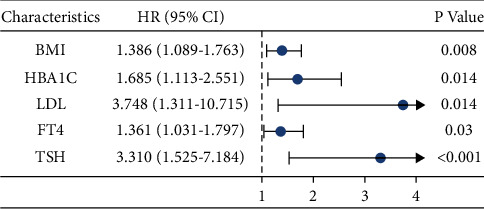
Logistic risk regression model: risk factors for nonalcoholic fatty liver disease (NAFLD).

A multivariate logistic regression model was used to analyze the risk factors for NAFLD. After adjusting for all factors with a *p*-value of <0.05, BMI, HbA1c, LDL, FT4, and TSH were identified as significant independent risk factors for NAFLD in the univariate analysis, with odds ratios of 1.386, 1.685, 3.748,1.361, and 3.310, respectively (all *p* < 0.05; Figure 1).

### 3.3. Visualization of the TH Signaling Pathway

**Figure 2:**
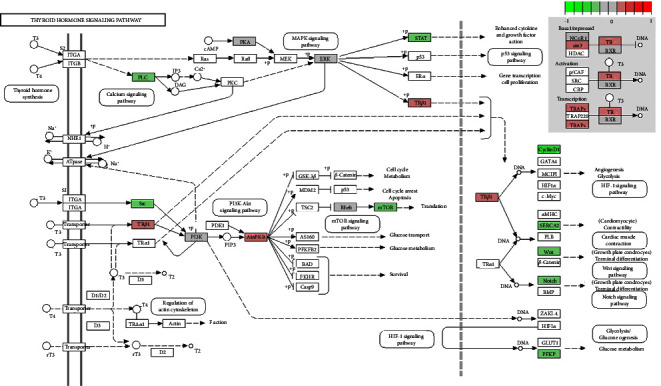
Changes in the expression of target genes of the thyroid hormone signaling pathway in patients with NAFLD. The changes are mapped with colors. The color depth positively correlated with the degree value. Red indicates increased expression, while green indicates decreased gene expression in the NAFLD group when compared with the healthy obese group.

Considering its important metabolic role, the liver may be the primary cause of TH resistance. To identify changes in the TH signaling pathway in patients with NAFLD, we downloaded the relevant expression profiles from the GSE48452 dataset. The Pathview library was used to visualize the TH signaling pathway.  Figure 2 shows several key molecular pathways affected by NAFLD-induced transcriptomic changes in liver samples. The results showed that the expression of thyroid hormone receptor beta (THRB), which encodes TR*β*, was significantly decreased in the NAFLD group, which may explain the TH resistance-like manifestation observed in the clinical findings.

### 3.4. Metabolic Characteristics of T2DM and NAFLD by Liraglutide Use

A total of 49 patients with T2DM and NAFLD received liraglutide treatment. The duration of liraglutide use ranged from 2 to 40 months. After liraglutide therapy (average duration of 16 months), these patients had significantly lower BMI, HbA1c, and TSH but higher HDL levels than the baseline (all *p* < 0.05). No significant differences in ALT, AST, alkaline phosphatase, r-glutamyl transferase, eGFR, LDL-c, FT3, and FT4 levels and the use of hypoglycemic drugs were observed between the two groups (all *p* > 0.05; Table 2).

### 3.5. Subgroup Analysis with Stratification by Duration of Liraglutide Use

**Figure 3:**
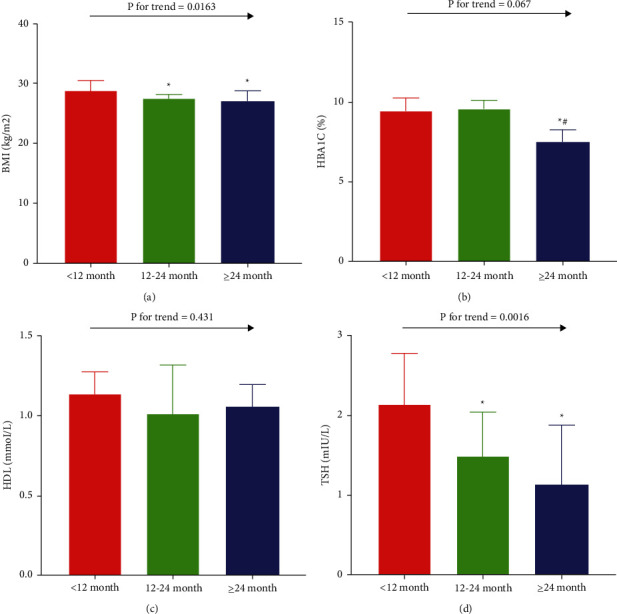
Subgroup analysis with stratification by usage period of liraglutide. Covariance analysis of (a) body mass index, (b) hemoglobin A1c, (c) high-density lipoprotein, and (d) thyroid-stimulating hormone (TSH). Values are expressed as the mean ± standard deviation. ^*∗*^*p* < 0.05 vs. the <12 months group; ^#^*p* < 0.05 vs. the ≥12 and <24 months group.

The time of liraglutide administration will have an effect on the abovementioned indicators. An additional analysis of covariance was performed to compare factors with significant associations based on univariate analysis. Patients were stratified into three groups according to the duration of liraglutide use: <12 months (*N* = 10), ≥12 and <24 months (*N* = 30), and ≥24 months (*N* = 9). The test for time trends showed statistical differences in BMI (Figure 3(a)) and TSH (Figure 3(d)) but not in HbA1c (Figure 3(b)) and HDL (Figure 3(c)). Significantly higher BMI and TSH levels were observed in the <12 months group than in the other two groups (both *p* < 0.05).

### 3.6. Concentration of TSH Stratified by Status of Liver Disease

**Figure 4:**
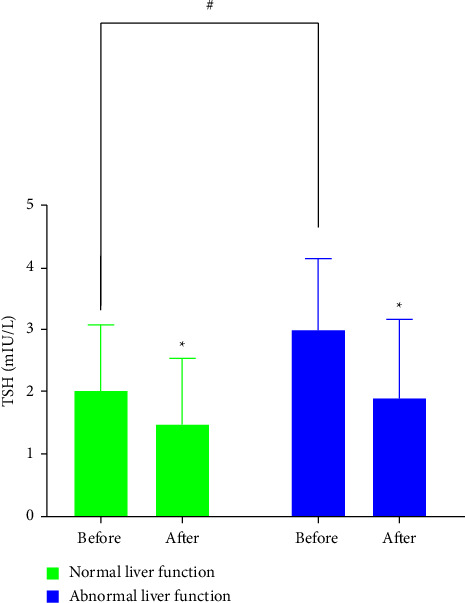
Concentration of TSH before and after liraglutide treatment stratified by status of liver disease. ^*∗*^*p* < 0.05 vs. corresponding baseline data; ^#^*p* < 0.05 vs. baseline normal liver function group.

As mentioned above, a clear downward trend in serum TSH levels was observed with extended use of liraglutide. Further analysis with stratification by status of liver disease was performed to estimate whether the status of liver disease at baseline is intrinsically related to the decrease in TSH levels. The status of liver disease was categorized into two groups as follows: normal liver function (*N* = 39) and abnormal liver function (*N* = 10), in which the ALT or AST level was above the normal reference range. At baseline, patients with abnormal liver function had significantly higher TSH levels than those with normal liver function. After liraglutide therapy, both groups showed significantly decreased TSH levels compared with the corresponding baseline data (both *p* < 0.05; Figure 4).

## 4. Discussion

In our clinical study, after adjusting for overweight/obese BMI and diabetes, the two most common causes of NAFLD, a manifestation of TH resistance, were found, suggesting that intrahepatic damage of the TH pathway may play an important role in the occurrence of NAFLD. Bioinformatics analysis indicated that THRB was significantly downregulated in obese patients with NAFLD, which may explain the abovementioned phenomenon. The manifestation of TH resistance improved significantly with liraglutide treatment, regardless of the status of the liver disease, and the level of TSH decreased in a time-dependent manner. Collectively, these findings indicate that liraglutide improves hepatic TH resistance in T2DM with NAFLD, and restoration of impaired TR*β* expression in NAFLD may be a potential mechanism involved in the process of liraglutide therapy.

NAFLD is a serious global health epidemic that causes a growing burden on public health [9]. The accumulation of liver fat, which is most commonly observed in cases of obesity or T2DM, may drive further disease progression [10]. Therefore, we designed a study for individuals diagnosed with T2DM and are overweight/obese, adjusting for the interference from metabolic dysfunction beyond the liver. In this study, multivariate analysis demonstrated that TSH and FT4 were significant independent risk factors for NAFLD, suggesting that intrahepatic damage of the TH pathway may play an important role in the occurrence of NAFLD independent of obese/overweight or T2DM conditions. TH has significant effects on lipid metabolism in the liver [11, 12]. NAFLD caused by hypothyroidism is usually attributed to interruption of TH signaling, resulting in decreased lipid utilization by the liver [13, 14]. Indeed, subclinical hypothyroidism was found to be associated with NAFLD in a dose-dependent manner, even within the upper range of normal serum TSH concentrations [15, 16]. There are two major isoforms of the thyroid hormone receptor (TR), TR*α* and TR*β*, which are the predominant receptors in the liver [17]. TH metabolites [18], TR*β* agonists [19], and liver-specific analogs [20] have been studied as potential therapeutics for treating both serum dyslipidemia and NAFLD. Resmetirom (MGL-3196) and Hep-Direct prodrug VK2809 (MB07811) may be two of the most promising lipid-lowering agents currently in phase 2-3 clinical trials [19, 21]. Chaves et al. demonstrated that impairments in liver TR*β* signaling due to THRB gene mutations can lead to hepatic steatosis, indicating the influence of TH on lipid metabolism in the liver [22]. Similarly, TR*β* impairment was also supported by bioinformatics analysis in our study, which emphasizes the important role of thyroid function in NAFLD, in addition to the metabolic contributions of diabetes and obesity. Despite encouraging results for the treatment of obesity, dyslipidemia, and liver cancer, serious adverse reactions have limited their use in clinical trials. Additionally, research on the association between TR*β* and NAFLD, which may involve the intrinsic mechanism of NAFLD development, remains insufficient.

Liraglutide is a GLP-1R agonist (GLP-1RA) that enhances meal-stimulated insulin secretion and reduces glucagon secretion, gastric emptying, intestinal lipoprotein secretion, food intake, and body weight, thereby improving the metabolic health of animals and humans [23, 24]. Data from animal studies and randomized human trials indicate reductions in liver fat, fibrosis, and inflammation after sustained GLP-1RA administration through mechanisms that have not been fully elucidated [25, 26]. At present, studies on GLP-1RA in the treatment of NAFLD mainly focus on lipid metabolism [27]. The effects of GLP-1 on *de novo* lipogenesis, *β*-oxidation, chylomicron import, and very low-density lipoprotein export collectively contribute to the lipid-lowering effects of GLP-1RA [28]. The vital effects of TH on liver metabolism and the TH resistance-like phenomenon have been demonstrated in the pathogenesis of NAFLD. In the present study, the manifestation of TH resistance improved significantly with liraglutide treatment in a time-dependent manner. Nonalcoholic steatohepatitis (NASH) is characterized by hepatic steatosis and inflammation, whereas NAFLD is a contemporary term for fatty liver disease associated with metabolic dysfunction [29]. Studies have shown that subclinical hypothyroidism and low-normal thyroid function are independent predictors of advanced fibrosis and NASH [30, 31]. GLP-1RA reduces liver fat and inflammation in rodent and human studies, supporting its investigational use in people with T2DM and NAFLD who are at risk for developing NASH [25, 32, 33]. In this study, the NAFLD and NASH groups showed significantly decreased TSH levels after liraglutide therapy compared with the corresponding baseline data, indicating that the manifestation of TH resistance improved significantly with liraglutide treatment regardless of the status of liver disease.

To the best of our knowledge, this study is the first to investigate the effect of liraglutide on serum TSH levels in patients with NAFLD. The results provide insights into NAFLD therapies. Despite these findings, our study has some limitations. First, the sample size was relatively small, and unmeasured confounders may not be fully solved; thus, the results may not be broadly applicable. Second, ultrasonography has limited sensitivity and does not reliably detect steatosis when its level is <20% or in individuals with high BMI (>40 kg/m^2^), which might have led to potential heterogeneity. Finally, the mechanisms by which TR*β* plays a role in the therapeutic process of liraglutide are not completely clear, and more evidence is required to determine the biological foundation.

In summary, liraglutide improves hepatic TH resistance in T2DM with NAFLD, and restoration of impaired TR*β* expression in NAFLD may be a potential mechanism involved in the process of liraglutide therapy.

## Figures and Tables

**Table 1:** Demographic and metabolic characteristics of study subjects.

Characteristic	Non-NAFLD (*N* = 49)	NAFLD (*N* = 49)	*p*
General			
Gender, *n* (%)			0.832
Male	33 (67.3%)	31 (63.3%)	
Female	16 (32.7%)	18 (36.7%)	
Age, year	57.22 ± 12.33	58.88 ± 12.89	0.431
Duration of diabetes, year	9 (4, 15)	6 (0, 10)	0.042
BMI, kg/m2	26.12 (25.08, 28.3)	28.76 (26.93, 30.9)	<0.001

Biochemical markers			
FBG, mmol/L	7.66 (6.38, 9.85)	9.99 (7.49, 12.88)	0.017
HbA1c, %	8 (6.8, 10.4)	10 (8.57, 11.4)	<0.001
LDL, mmol/L	2.25 ± 0.74	2.68 ± 0.87	0.012
HDL, mmol/L	0.96 (0.81, 1.1)	0.84 (0.72, 0.98)	0.009
ALT, IU/L	22 (17, 39)	25 (18, 42)	0.384
AST, IU/L	23 (17, 29)	21 (15, 33)	0.440
eGFR, ml/min/1.73 m^2^	115.23 ± 51.74	121.59 ± 45.18	0.154
FT3, pmol/L	5.03 (4.52, 5.34)	4.76 (4.34, 5.28)	0.238
FT4, pmol/L	13.03 (11.33, 13.79)	14.17 (12.49, 16.07)	0.005
TSH, mIU/L	1.26 (0.9, 1.71)	1.94 (1.12, 2.95)	0.004

Use of hypoglycemic drugs			
Insulin (N, %)	20 (40.8%)	21 (42.9%)	0.838
Metformin (N, %)	28 (57.1%)	29 (59.2%)	0.838
Thiazolidinediones (N, %)	2 (4.1%)	3 (6.1%)	0.646
Sulfonylureas/glinides (N, %)	3 (6.1%)	3 (6.1%)	1.000
Glucosidase inhibitors	11 (22.4%)	11 (22.4%)	1.000
SGLT2 inhibitors	21 (42.9%)	29 (59.2%)	0.106

**Table 2:** Metabolic characteristics of patients with type 2 diabetes and nonalcoholic fatty liver disease by liraglutide use.

Characteristic	Before	After	*p*
General and biochemical markers	49	49	
BMI, kg/m2	28.76 (26.93, 30.9)	27.72 (26.79, 30.85)	0.011
FBG, mmol/L	9.99 (7.49, 12.88)	8.13 (6.57, 11.30)	0.496
HbA1c, %	10 (8.57, 11.4)	8.7 (7.8, 9.55)	0.009
LDL, mmol/L	2.68 ± 0.87	2.34 ± 0.80	0.433
HDL, mmol/L	0.84 (0.72, 0.98)	0.96 (0.81, 1.088)	0.023
ALT, IU/L	25 (18, 42)	24 (15, 37)	0.332
AST, IU/L	21 (15, 33)	18 (16.26.2)	0.210
ALP, IU/L	81.41 ± 30.19	77.33 ± 15.5	0.244
r-GT, IU/L	32.35 (21.25, 67.78)	34.6 (19, 42)	0.856
eGFR, ml/min/1.73 m^2^	115.23 ± 51.74	109.96 ± 31.74	0.102
FT3, pmol/L	4.76 (4.34, 5.28)	4.51 (4.18, 5.11)	0.826
FT4, pmol/L	14.17 (12.49, 16.07)	13.24 (11.92, 14.47)	0.975
TSH, mIU/L	1.94 (1.12, 2.95)	1.46 (0.89, 2.04)	0.019

Use of hypoglycemic drugs			
Insulin (N, %)	21 (42.9%)	18 (36.7%)	0.536
Metformin (N, %)	29 (59.2%)	34 (69.4%)	0.292
Thiazolidinediones (N, %)	3 (6.1%)	1 (2.0%)	0.617
Sulfonylureas/glinides (N, %)	3 (6.1%)	1 (2.0%)	0.617
Glucosidase inhibitors	11 (22.4%)	7 (14.3%)	0.545
SGLT2 inhibitors	29 (59.2%)	27 (55.1%)	0.683

## Data Availability

The datasets analyzed during the current study are available from the corresponding author.
